# Simcluster: clustering enumeration gene expression data on the simplex space

**DOI:** 10.1186/1471-2105-8-246

**Published:** 2007-07-11

**Authors:** Ricardo ZN Vêncio, Leonardo Varuzza, Carlos A de B Pereira, Helena Brentani, Ilya Shmulevich

**Affiliations:** 1 Institute for Systems Biology, 1441 North 34th street, Seattle, WA 98103-8904, USA; 2 BIOINFO-USP – Núcleo de Pesquisas em Bioinformática, Universidade de São Paulo, São Paulo, Brazil; 3 Hospital do Câncer A. C. Camargo, São Paulo, Brazil

## Abstract

**Background:**

Transcript enumeration methods such as SAGE, MPSS, and sequencing-by-synthesis EST "digital northern", are important high-throughput techniques for digital gene expression measurement. As other counting or voting processes, these measurements constitute compositional data exhibiting properties particular to the simplex space where the summation of the components is constrained. These properties are not present on regular Euclidean spaces, on which hybridization-based microarray data is often modeled. Therefore, pattern recognition methods commonly used for microarray data analysis may be non-informative for the data generated by transcript enumeration techniques since they ignore certain fundamental properties of this space.

**Results:**

Here we present a software tool, Simcluster, designed to perform clustering analysis for data on the simplex space. We present Simcluster as a stand-alone command-line C package and as a user-friendly on-line tool. Both versions are available at: http://xerad.systemsbiology.net/simcluster.

**Conclusion:**

Simcluster is designed in accordance with a well-established mathematical framework for compositional data analysis, which provides principled procedures for dealing with the simplex space, and is thus applicable in a number of contexts, including enumeration-based gene expression data.

## Background

Technologies for high-throughput measurement of transcriptional gene expression are mainly divided into two categories: those based on hybridization, such as all microarray-related technologies [1,2] and those based on transcript enumeration, which include SAGE [3], MPSS [4], and Digital Northern powered by traditional [5] or, recently developed, EST sequencing-by-synthesis (SBS) technologies [6].

Currently, transcript enumeration methods are relatively expensive and more time-consuming than methods based on hybridization. However, recent improvements in sequencing technology, powered by the "$1000 genome" effort [7], promises to transform the transcript enumeration approach into a fast and accessible alternative [8-10] paving the way for a systems-level absolute digital description of individualized samples [11].

Methods for finding differentially expressed genes have been developed specifically in the context of enumeration-based techniques of different sequencing scales such as EST [12], SAGE [13] and MPSS [14]. However, in spite of their differences, hybridization-based and enumeration-based data are typically analyzed using the same pattern recognition techniques, which are generally imported from the microarray analysis field.

In the case of clustering analysis of gene profiles, the simple appropriation of practices from the microarray analysis field has been shown to lead to suboptimal performance [15]. Cai and co-workers [15] provided an elegant clustering computational solution to group tag (rows in a usual expression matrix representation) profiles that takes into account the specificities of enumeration-based datasets. However, to the best of our knowledge, a solution for transcript enumeration libraries (columns in a usual expression matrix representation) is still needed. We report on a novel computational solution, called Simcluster, to support clustering analysis of transcript enumeration libraries.

## Implementation

### Theory

Without loss of generality, we use the term "tag" to refer to the transcripts' representation, as usual in the SAGE field (this is equivalent to the term "signature" in MPSS analysis or "contigs" in EST analysis). The theoretical model used here to describe the transcript enumeration process is the usual uniform sampling of interchangeable colored balls from an infinite urn model. Given the total number *n *of counted tags and the abundance vector ***π ***of all transcripts, this model leads to a probabilistic description of the observed result: ***x***|***π***, *n *~ Multi(***π***, *n*), i.e., the counts ***x ***follow a Multinomial distribution [16]. It is also possible to model ***x ***as Poisson distributed [17] since it is an approximation for the Multinomial. Regardless of the specificities of the theoretical probabilistic model, it is well known that, as with other counting or voting processes, the natural space for dealing with this kind of data is the simplex space. The unitary simplex space, having *d *dimensions, is defined as [18,19]:

(1) $$S_{d-1}=\{\pi |\pi \in {\mathbb{R}}_{+}^{d},\pi 1^{\prime }=1\}$$

where **1 **is a vector of ones. In the gene expression context, *d *is the number of unique tags observed. An example of a simplex vector is ***p ***= E[***π***|***x***] and applying a standard Bayesian approach, one obtains from ***x***|***π***, *n*, using a Dirichlet prior density ***π ***~ Dir(***α***), the posterior density: ***π***|***x ***~ Dir(***x ***+ ***α***).

It is known that clustering analysis is inherently dependent on the choice of a distance measure between the considered objects. This, in turn, is connected to the structure of the underlying space. A metric Δ, measuring the distance between two objects *a *and *b*, must respect the properties:

(i) Δ(*a, b*) = Δ(*b, a*);

(ii) Δ(*a, b*) = 0 ⇔ *a *= *b*;

(iii) Δ(*a, c*) ≤ Δ(*a, b*) + Δ(*b, c*).

One may also consider additional reasonable properties such as:

(iv) scale invariance Δ(*xa, yb*) = Δ(*a, b*), *x, y *∈ ℝ_+_; and

(v) translational invariance Δ(*a *+ *t, b *+ *t*) = Δ(*a, b*).

These commonly required additional properties guarantee that distance measurements are not affected by the definition of arbitrary scale or measurement units and that more importance is given to the actual difference between the objects being measured rather than commonalities (more details can be found in the appendix Additional File 1).

Translations on the simplex space are defined by [19]:

(2) $$p⊕t=\frac{\left(p\cdot t\right)}{(p\cdot t)1^{\prime }}$$

where · is the usual Hadamard product and the division is vector-evaluated.

Well known distances, such as Euclidean, Manhattan, and correlation-based distances, do not exhibit the properties (i)-(v) if the measured objects belong to the simplex space, as is the case of transcript enumeration data. A possible metric that obeys (i)-(v) on the simplex space is the Aitchisonean distance [19]:

(3) $$\Delta (p,q)=\sqrt{ln\left(\frac{p_{-d}/p_{d}}{q_{-d}/q_{d}}\right){\left(I+1^{\prime }\times 1\right)}^{-1}ln{\left(\frac{p_{-d}/p_{d}}{q_{-d}/q_{d}}\right)}^{\prime }}$$

where ***I ***is the identity matrix, × is the Kronecker product, -*d *subscript is a notation for "excluding the *d*^*th *^element", and elementary operations are vector-evaluated.

Clustering procedures coherent with this theoretical background are suitable for transcript enumeration data.

### Software design

In short, Simcluster's method can be described as the use of a Bayesian inference step (currently with a uniform prior) to obtain the expected abundance simplex vectors given the observed counts E[***π***|***x***], and the use of the Aitchisonean distance in the following algorithms: k-means, k-medoids and self-organizing maps (SOM) for partition clustering, PCA for inferring the number of variability sources present, and common variants of agglomerative hierarchical clustering.

Currently, the Simcluster package is comprised of: Simtree, for hierarchical clustering; Simpart, for partition clustering; Simpca for Principal Component Analysis (PCA); and several utilities such as TreeDraw, a program to draw hierarchical clustering dendrograms with user-defined colored leaves. Simcluster's modularity allows relatively simple extension and addition of new modules or algorithms. Increasing the coverage of algorithms and validity assessment methods [20] are envisioned in future updates. Simcluster can be used, modified and distributed under the terms of the GPL license [21]. The software was implemented in C for improved performance and memory usage, assuring that even large datasets can be processed on a regular desktop PC (Additional File 2).

To increase source code reuse, established libraries were used: Cluster 3 [22] for clustering, GNU Scientific library [23] for PCA, Cairo [24] and a modification of TreeDraw X [25] for colored dendrogram drawing. The input data set can be a matrix of transcript counts or general simplex vectors. Some auxiliary shell and Perl scripts are available to: automatically download data from the GEO database [26], convert GEO files to Simcluster input format, and filter out low-count tags.

The Linux-based installation and compilation is facilitated by a configuration script that detects all the prerequisites for Simcluster compilation. Missing libraries are automatically downloaded from the Simcluster website and compiled by the Simcluster compilation process.

**Figure 1:**
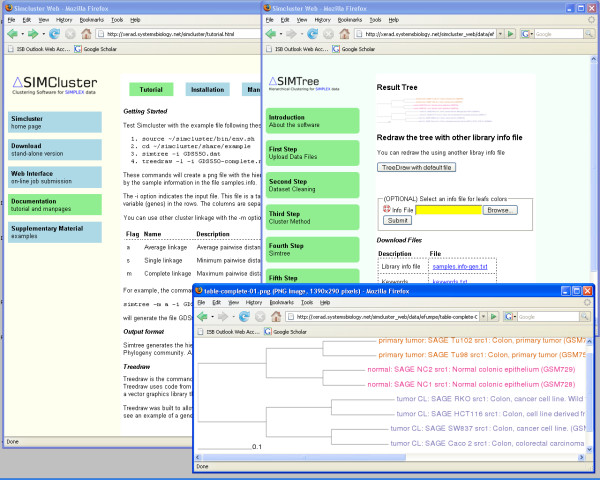
**Screenshot of an analysis session using Simcluster's web-based interface**. Simcluster's on-line version was designed to be a user-friendly interface for the command-line version. The screenshot shown is an illustration of an interactive session usign the example data provided.

To broaden usability, a user-friendly web interface was developed and is made available at http://xerad.systemsbiology.net/simcluster_web/. Figure 1 shows a screenshot of an analysis session using Simcluster's web-based interface.

## Results and Discussion

We agree with Dougherty and Brun [27,28] that "validation" of clustering results is a heuristic process, even though there are some interesting efforts to objectively incorporate biological knowledge in this process using Gene Ontology, especially when one is clustering gene expression profiles [29,30]. However, to illustrate the usefulness of our software, we collected several examples in which the performance of Simcluster can be considered as qualitatively superior to some traditional approaches imported from the microarray analysis field. These examples include EST, SAGE and MPSS datasets, and are available on the project's webpage [31]. Among these, we describe here a simulated enumeration dataset built from real microarray data, for which we can define the ground truth and check results against it in a relatively objective way. Of course, a comprehensive study with simulated data, consisting of comparisons of clustering algorithms, distance metrics, and distributions generating the random point sets, would be necessary to properly evaluate any clustering algorithm. This should be the subject of future work. The objective of this example is to show that Simcluster is able to reconstruct the clustering result obtained for an Affymetrix microarray dataset when the input is a simulated transcript enumeration dataset, built to mimic the real microarray biological data.

The data used to create the virtual transcript enumeration data was obtained from the Innate Immunity Systems Biology project [32] and is provided as an Additional File 3. This data is a set of Affymetrix experiments of mouse macrophages stimulated by different Toll-like receptor agonists (LPS, PIC, CPG, R848, PAM) during a time-course (0, 20, 40, 60, 80 and 120 minutes). A detailed description and biological significance of this dataset is presented elsewhere [32,33].

**Figure 2:**
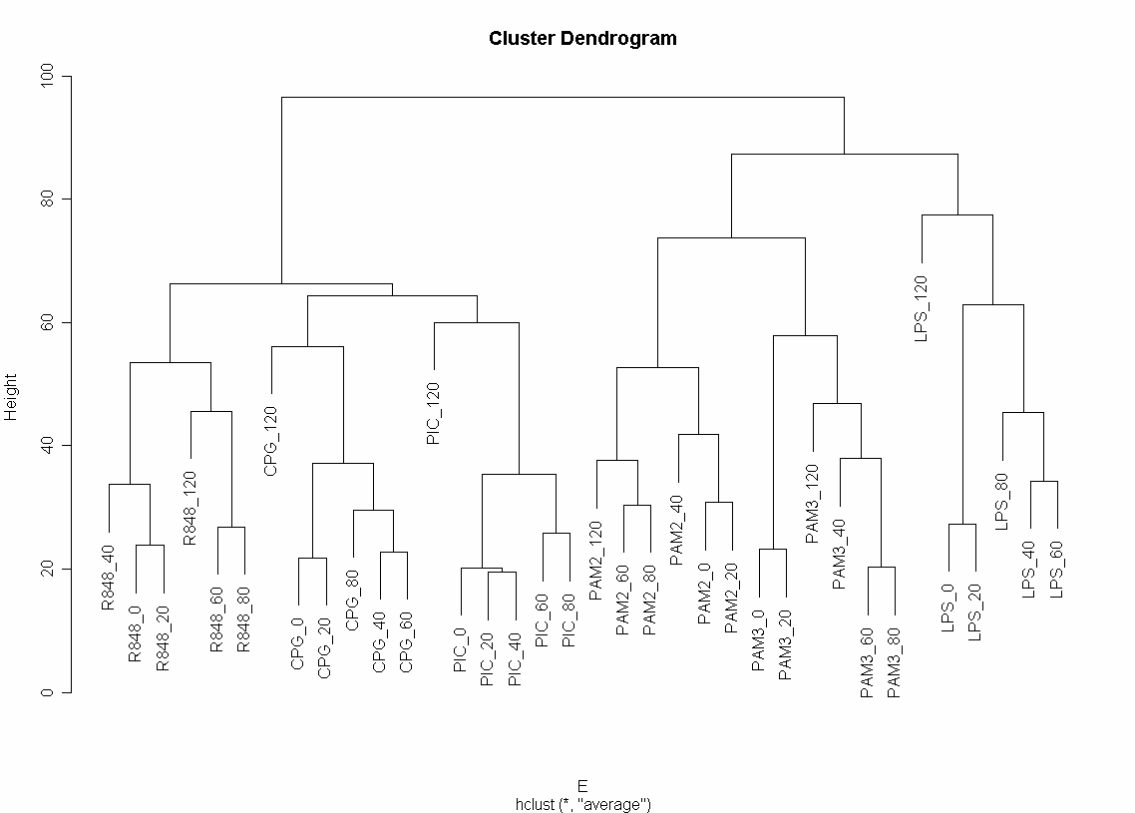
**Clustering analysis of the Affymetrix dataset**. Data produced by the Innate Immunity Systems Biology project [32,33] and available as Additional File 3. This data is a set of Affymetrix experiments of mouse macrophages stimulated by different Toll-like receptor agonists (LPS, PIC, CPG, R848, PAM) during a time-course (0, 20, 40, 60, 80 and 120 minutes). Method: Euclidean distance with average linkage agglomerative hierarchical clustering.

Using this data, a clustering analysis result is shown in Figure 2. This pattern is obtained using the most common type of clustering analysis in the microarray field: Euclidean distance with average linkage agglomerative hierarchical clustering, implemented by R [34] routines, available as Additional File 3. This clustering pattern will be considered to be the "gold-standard" for the purpose of this simulation.

The virtual experiment consists of the creation of a transcriptome with the relative abundance between genes defined by the Affymetrix data; sampling a random number of tags from it of different magnitudes; enumeration of sampled transcripts; and using some common clustering procedures along with Simcluster. It is easier to understand the concept of the virtual transcriptome by following a particular case. For the sample labeled LPS-120 measured 120 minutes after the LPS stimulus, for the Affymetrix expression levels see Table 1.

**Table 1:** 

Probesets	Representative ID	Gene Symbol	Intensity (sorted)
1457375_at	BG094499	Transcribed locus	1.94760
1452109_at	BG973910	interleukin 17 receptor E	2.14522
...	...	...	...
M12481_3_at	AFFX-b-ActinMur	actin beta cytoplasmic	36191.41765
1436996_x_at	AV066625	P lysozyme structural	43458.17590

The virtual total number of available tags is defined as proportional to the measured intensity using 10,000 as a scaling constant, an arbitrary number large enough to assure that finite population issues are negligible. Actual examples are: 19,476 for BG094499; 21,452 for BG973910; and so on until 361,914,176 for actin; and 434,581,759 for AV066625. The total amount of available tags is *T *= 126,971,909,452, which is a number much greater than the typical number of sequenced tags and is in accordance with the "infinite urn" model.

**Figure 3:**
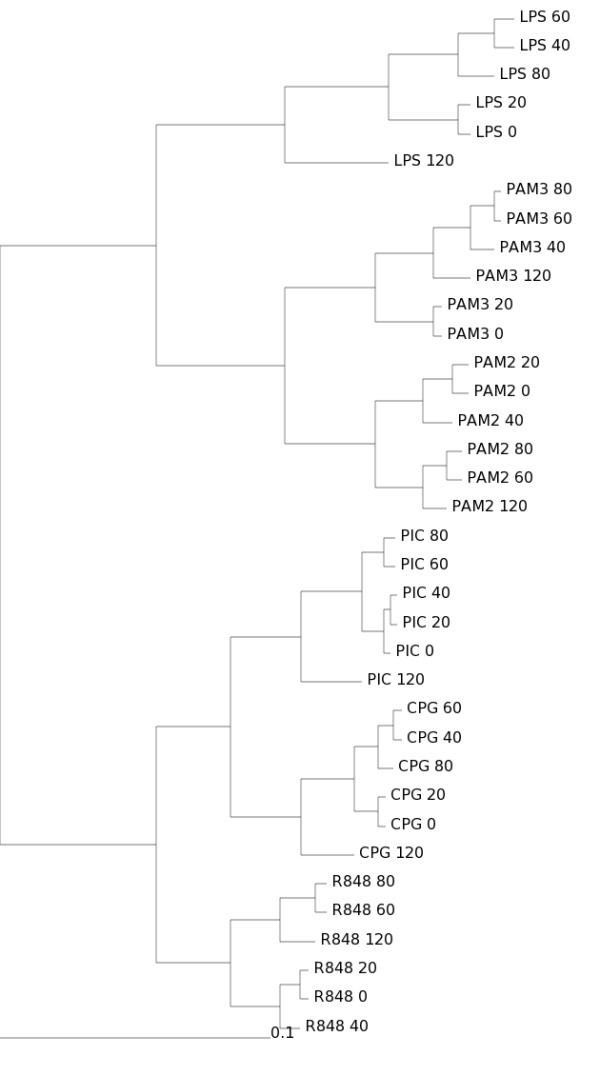
**Simcluster's clustering of simulated data based on Affymetrix expression levels**. Transcript enumeration data produced by the simulation of a virtual transcriptome according to the Affymetrix expression levels. Sample size *n *= 100,000,000. Method: Simcluster's average linkage agglomerative hierarchical clustering.

**Figure 4:**
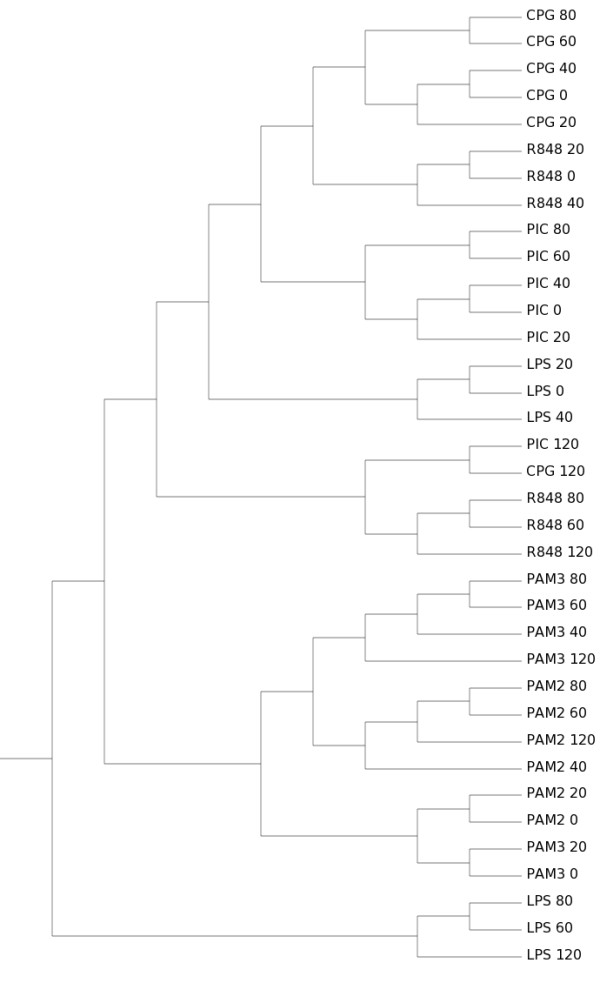
**Clustering of simulated data using Euclidean distance**. Transcript enumeration data produced by the simulation of a virtual transcriptome according to the Affymetrix expression levels. Sample size *n *= 100,000,000. Method: Euclidean distance with average linkage agglomerative hierarchical clustering.

**Figure 5:**
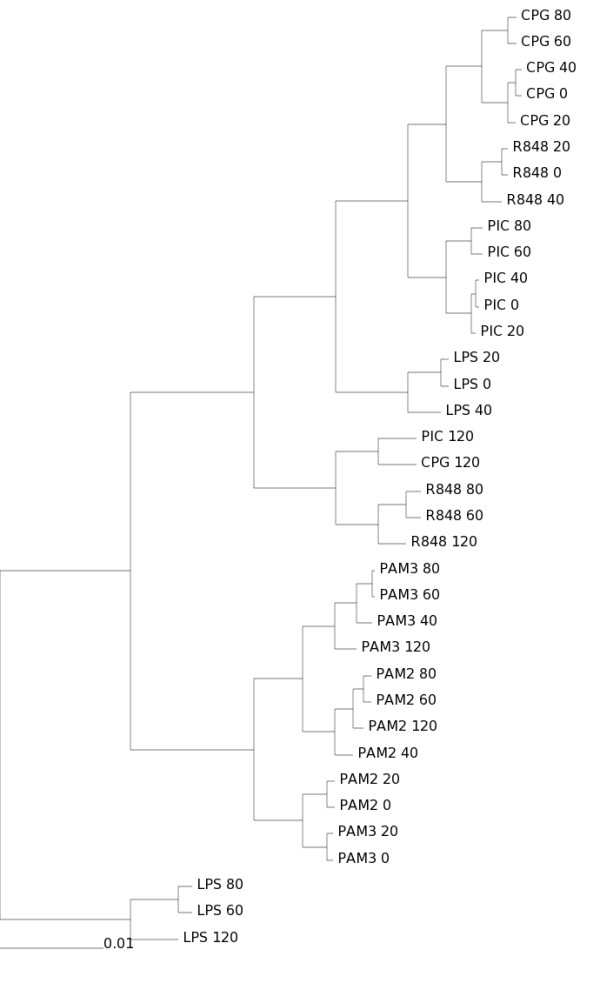
**Clustering of simulated data using correlation distance**. Transcript enumeration data produced by the simulation of a virtual transcriptome according to the Affymetrix expression levels. Sample size *n *= 100,000,000. Method: correlation-based distance with average linkage agglomerative hierarchical clustering.

**Figure 6:**
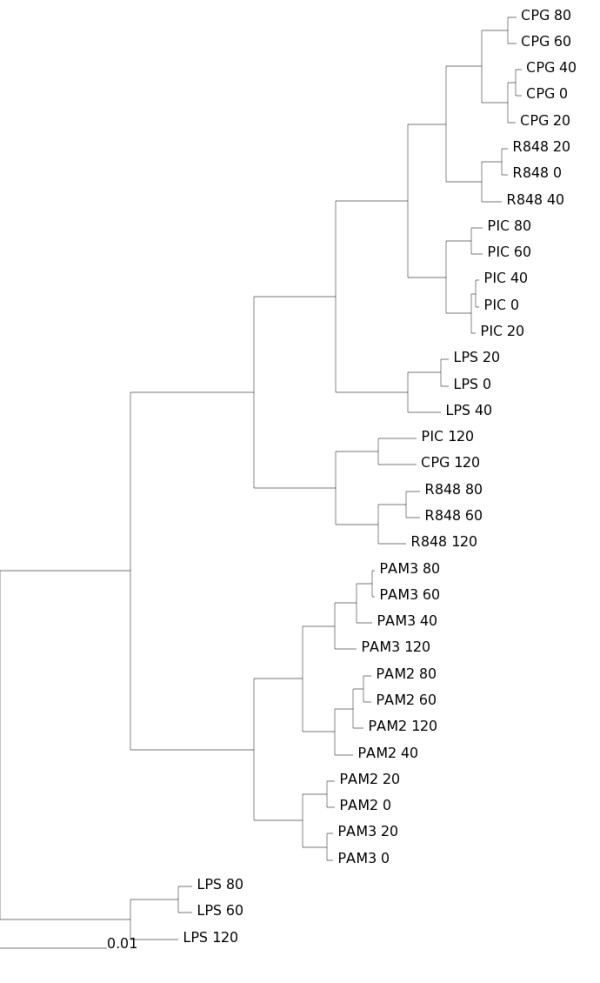
**Clustering of simulated data using cosine distance**. Transcript enumeration data produced by the simulation of a virtual transcriptome according to the Affymetrix expression levels. Sample size *n *= 100,000,000. Method: cosine distance with average linkage agglomerative hierarchical clustering.

The total of virtually sequenced tags *N *for each sample is simulated from a Poisson distribution, *N *~ Poisson(*n*), to create a realistic virtual sequencing library. All generated data and results are available as Additional File 3. For example, the actual simulation for *n *= 1,000,000 virtually sequenced tags assigned *N *= 1,001,794 for the LPS-120 library; *N *= 998,382 for the CPG-40 library; and so on. The same process is repeated for increasing *n *from 100,000 to 100,000,000. Since *n *≪ *T *for all *n *considered, the multinomial sampling is used and its mean is taken for each library, according to the assumed "infinite urn" model. The results for the largest simulation are shown in Figures 3, 4, 5, 6 and individual results for all separate increasing *n *sizes are available as Additional File 3.

It is clear that cluster results obtained by Simcluster converge to the same structure obtained by analyzing the Affymetrix data, as the number of virtually sequenced tags increases. Moreover, Simcluster's results are not only compatible with the usual microarray analysis for Affymetrix data, but also are more biologically meaningful than the results obtained by the usual microarray analysis techniques applied to the virtual sequencing data. As in the original microarray analysis, the Simcluster result is able to cluster together the different stimuli, placing consecutive time-points close to each other.

Although this kind of analysis certainly does not provide a proof, the above result indicate that the theoretical framework is adequate for enumeration-based data, as expected. Additional examples and discussions can be found on the project's website [31].

## Conclusion

We developed a software tool, called Simcluster, for clustering libraries of enumeration-based data. It is important to note that Simcluster is built in accordance with a well-established mathematical framework for compositional data analysis, which provides principled procedures for dealing with the simplex space, and is thus applicable in contexts other than transcript enumeration.

## Availability and requirements

• Project Name: Simcluster

• Project Home Page: http://xerad.systemsbiology.net/simcluster

• Operating Systems: Linux for the stand-alone version and platform independent for the web-based tool.

• Programming Languages: C for the stand-alone version and C, Perl and HTML for the web-based tool.

• Other requirements: some GNU/GPL or GNU/LGPL libraries distributed together with the main package.

• License: GNU General Public License 2.0

## List of abbreviations

EST – Expressed Sequence Tag

SAGE – Serial Analysis of Gene Expression

MPSS – Massive Parallel Signature Sequencing

SBS – Sequencing-By-Synthesis

## Authors' contributions

RZNV proposed and conducted the study. LV wrote the software and helped to interpret the results. CABP indicated the compositional analysis literature and is LV's PhD thesis advisor. HB provided biological insight for result interpretation. IS supervised the study. RZNV and IS wrote the manuscript. All authors read and approved the final manuscript.

## Supplementary Material

Additional file 1 **Appendix: Aitchisonean distance**. Contains an appendix with some background on the usage of the Aitchisonean distance. Click here for file

Additional file 2 **Stand-alone command-line Simcluster version 0.8.14**. Simcluster version used to create results of this work. Note that this version is distributed for compatibility issues only and users should always obtain the latest version at the project's website: http://xerad.systemsbiology.net/simcluster. Click here for file

Additional file 3 **Simulation data, results and scripts**. Contains the script that generated the virtual transcript enumeration data, the dataset used as the basis for the analysis, the results from it, and the conclusions for all tested samples sizes *n *from 100,000 to 100,000,000. Click here for file
