# Supplementary material for: Simcluster: clustering enumeration gene expression data on the simplex space
Source: BMC Bioinformatics. 2007 Jul 11;8:246. doi: 10.1186/1471-2105-8-246 (PMC2147035; doi:10.1186/1471-2105-8-246)
Supplement: Additional file 1 — Appendix: Aitchisonean distance. Contains an appendix with some background on the usage of the Aitchisonean distance. [file 1471-2105-8-246-S1.pdf]

Appendix - Simcluster: clustering enumeration gene expression data on the simplex space

June 27, 2007

The aim of this appendix is to provide more background and intuition, without exceedingly mathematical rigour or formalism on the Aitchisonean distance (also known as Aitchison distance).

The Aitchisonean distance [1, 2] is a metric to measure distances between objects defined in the simplex space. The unitary simplex space, having d dimensions, is defined as [1, 2]:

$$S_{d-1} = \{\boldsymbol{\pi} | \boldsymbol{\pi} \in \mathbb{R}_{+}^{d}, \pi_1 + \cdots + \pi_d = 1\} \tag{1}$$

In words, this means that a vector in the simplex space is a vector of positive numbers (zero is not allowed in this definition) that must sum up to 1. A natural example would be a (non-null) distribution of probabilities $P_1 + \cdots + P_d = 1$ for an event with d possible outcomes. Other clear examples would be the percentages of votes that several candidates receive in an election or the mineral composition of a rock. This kind of data is known as compositional data and its statistical analysis presents a number of practical and theoretical challenges [1, 2, 3].

The study of gene expression using quantitative transcript sequencing or transcript enumeration is also one example of data that can be considered in the context of compositional data analysis. The unitary simplex space (eq. 1 above) is perhaps the most well known option, but the imposition of any constant other than 1 would be sufficient to define a simplex space.

It is known that clustering analysis, the main subject of the manuscript, is inherently dependent on the choice of a distance measure between the considered objects. This, in turn, is connected to the structure of the underlying space.

The simplex space is not like the tridimensional physical space that we are used to in our ordinary life. In fact, Einstein showed that our physical space is not actually a simple tridimensional space, as our intuition may indicate, but is actually a four-dimensional space where space and time are conected. Therefore, to measure physical distance between objects in astronomical scales one would not use the regular Euclidean distance:

$$\Delta(\boldsymbol{a},\boldsymbol{b}) = \sqrt{(x_a - x_b)^2 + (y_a - y_b)^2 + (z_a - z_b)^2} \tag{2}$$

but rather use proper relativisitc distance measurements. This complication arises because our world is not an Euclidean world.

As an intuitive example, consider the distance between two points on a spherical surface with unitary radius (figure 1), a non-Euclidean space. It is not meaningful to use the Euclidean distance, but rather:

$$\Delta(\boldsymbol{a},\boldsymbol{b}) = 2\arcsin\left(\sqrt{\sin^2\left(\frac{\phi_b - \phi_a}{2}\right) + \cos\phi_a \cos\phi_b \sin^2\left(\frac{\lambda_b - \lambda_a}{2}\right)}\right) \tag{3}$$

, where ϕ and λ the are points' latitude and longitude, respectively.

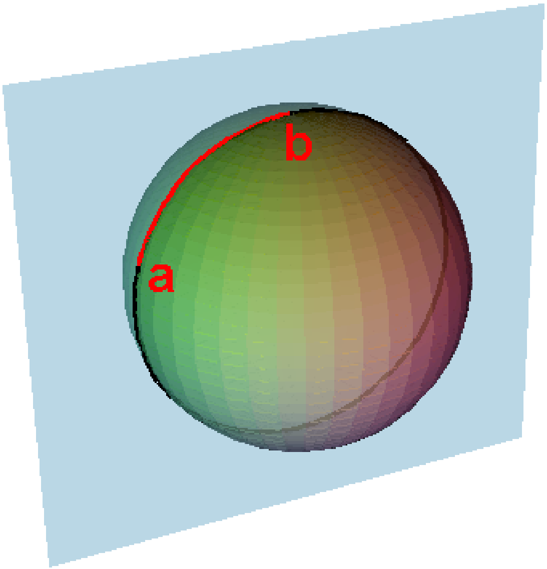

Figure 1: The distance between two points a and b in a spherical surface space is not measured by the Euclidean distance.

The same kind of situation is present when one is considering elements that belong to the simplex space, though the latter can be more difficult to visualize geometrically then the previous example. To measure objects in the simplex space, a meaningful distance was proposed by John Aitchison [2]:

$$\Delta(\boldsymbol{a},\boldsymbol{b}) = \sqrt{ln\left(\frac{\boldsymbol{a}_{-d}/a_d}{\boldsymbol{b}_{-d}/b_d}\right)(\boldsymbol{I}+\boldsymbol{1}'\times\boldsymbol{1})^{-1}\, ln\left(\frac{\boldsymbol{a}_{-d}/a_d}{\boldsymbol{b}_{-d}/b_d}\right)'} \tag{4}$$

where $\boldsymbol{I}$ is the identity matrix, $\times$ is the Kronecker product, the $-d$ subscript is a notation for "excluding the dth element", and elementary operations are vector-evaluated.

The compact form of the Aitchisonean distance presented in the equation 4 can be expanded noting that:

$$\boldsymbol{I}+\boldsymbol{1}'\times\boldsymbol{1} = \begin{pmatrix} 1 & 0 & 0 & \cdots & 0 \\ 0 & 1 & 0 & \cdots & 0 \\ & & \cdots & & \\ 0 & 0 & 0 & \cdots & 1 \end{pmatrix} + \begin{pmatrix} 1 & 1 & 1 & \cdots & 1 \\ 1 & 1 & 1 & \cdots & 1 \\ & & \cdots & & \\ 1 & 1 & 1 & \cdots & 1 \end{pmatrix}$$

and $ln\left(\frac{\boldsymbol{a}_{-d}/a_d}{\boldsymbol{b}_{-d}/b_d}\right) = (ln(a_1/a_d) - ln(b_1/b_p), \cdots, ln(a_{d-1}/a_d) - ln(b_{d-1}/b_p))$.

Using the expressions above, Δ can be re-arranjed as:

$$\Delta(\boldsymbol{a}, \boldsymbol{b}) = \frac{1}{d}\sqrt{\sum_{i<j}(ln(a_i/a_j) - ln(b_i/b_j))^2} \tag{5}$$

It is possible to show that some of the commonly used distances are special cases of this type of distance under particular scenarios. One can manipulate the simplex space with non-linear transformations in such a way that the Aitchisonean distance would be equivalent to the Euclidian distance in this new transformed ("stretched", "unfolded" or "inflated") space [2].

The Aitchisonean distance in the simplex space obeys the necessary requirements for a metric as well as some intuitive additional properties that are violated by other usual metric and non-metric measures, such as Euclidean or correlation-based distances.

A metric Δ, measuring the distance between two objects a and b, must respect the properties:

(i) $\Delta(a, b) = \Delta(b, a)$;

(ii) $\Delta(a, b) = 0 \Leftrightarrow a = b$;

(iii) $\Delta(a, c) \leq \Delta(a, b) + \Delta(b, c)$.

These properties are directly linked to our intuitive perception of physical space. The property (i) encodes the notion that the distance betwen two objects should not depend on the direction used to measure it. The property (ii) can be stated as the "identity of indiscernibles". The property (iii) is the well-known triangle inequality, which states that the shortest distance between two points is a straight "line". Note that using (i)-(iii), one can show that a distance cannot be negative: $\Delta(a, b) \geq 0$.

Of course, it is possible to selectively violate these properties, creating functions such as pseudometrics, hemimetrics or quasimetrics, depending on

which properties are dropped. One distance measurement, well known to the gene expression community, is the correlation distance, which is not a metric as it does not obey (i)-(iii).

When dealing with compositional data, it is reasonable to demand several additional features from a metric, such as:

(iv) scale invariance $\Delta(xa, yb) = \Delta(a, b), x, y \in \mathbb{R}_+$; and

(v) translational invariance $\Delta(a + t, b + t) = \Delta(a, b)$.

In pattern recognition, well-known methods such as k-means clustering or self-organizing maps (SOM), implemented in Simcluster, do not behave as expected if the scale invariance cannot be guaranteed. A classical example of misbehavior is when one obtains different clustering patterns of the same data depending on the measurement units, such as in figure 2.

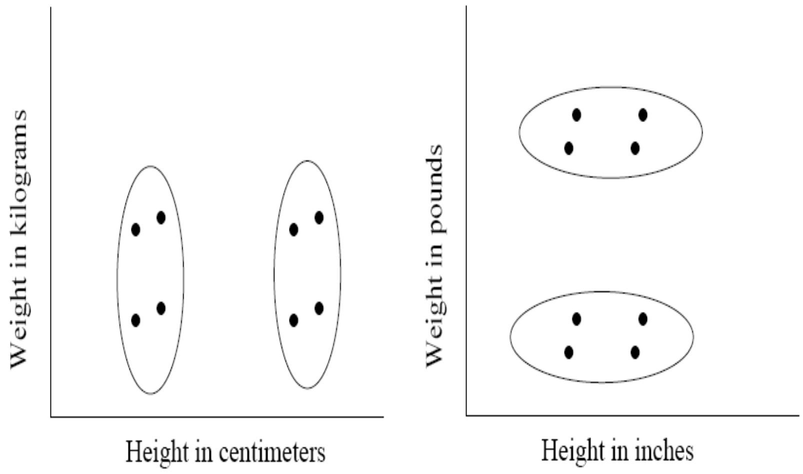

Figure 2: Clusters obtained using k-means on height and weight measurements of people (example obtained from [4])

Its implications can be noticed not only for transcript enumeration data, but also for compositional data in general. In the transcript enumeration field in particular, one can verify that there are several research groups that report

their results in different “units”. One such example is the molecules/cell unit, a rough estimate based on the estimated number of molecules N in a cell that, therefore, makes the total number of transcripts sum up to N. Another example is the transcript per million (TPM) unit, an arbitrary choice yielding a sum up of 1,000,000. Clearly, the analysis results should not depend on the measurement units, especially when all of them are linearly related. These desired features are incentives for the adoption of a scale invariant metric.

Intuitive justifications can be also seen for the use of metrics presenting the translational invariance property (v). When such a property is imposed, one is seeking to retain the intuitive properties of the Euclidean space. It would be desireable to have a measure of distance that does not depend on the particular position in the space but rather depends only on the objects being measured (figure 3).

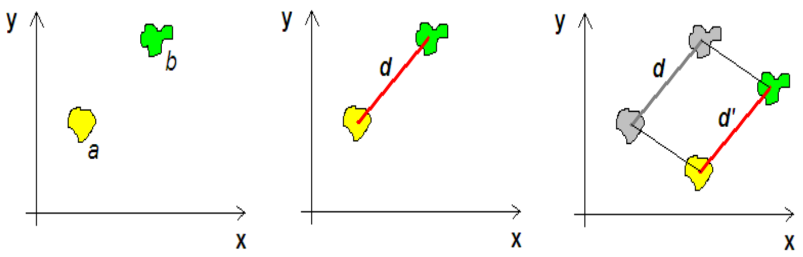

Figure 3: The distance d between objects a and b does not depend on the particular position in the space where the measurement is taken ($d' = d$).

The theory developed by John Aitchison to deal with the simplex space is complex. It is a complete and formal mathematical theory from which we just used a small part: the definition of distance between objects. However, once one accepts it as an appropriate way to describe objects in the simplex space obtained from compositional data such as transcript enumeration (and many others outside the field of Biology), it provides a simple recipe to measure distances and, therefore, an adequate framework for clustering analysis of this kind of data.

References

[1] J. Aitchison. *The Statistical Annalysis of Compositional Data*. Monographs on Statistics and Applied Probability. Chapman and Hall, London, 1986.

[2] J. Aitchison. Simplicial inference. In M.A.G. Viana and D.St.P. Richards, editors, *Algebraic Methods in Statistics and Probability: Contemporary Mathematics Series*, number 287 in Contemporary Mathematics Series, chapter 1, pages 1–22. American Mathematical Society, Providence, Rhode Island, 2001.

[3] J. Aitchison and JJ Egozcue. Compositional Data Analysis: Where Are We and Where Should We Be Heading? *Mathematical Geology*, 37(7), 2005.

[4] M. Kumar and J.B. Orlin. Scale-invariant Clustering with Minimum Volume Ellipsoids.
