# Supplementary figures and images for: Simcluster: clustering enumeration gene expression data on the simplex space
Source: BMC Bioinformatics. 2007 Jul 11;8:246. doi: 10.1186/1471-2105-8-246 (PMC2147035; doi:10.1186/1471-2105-8-246)

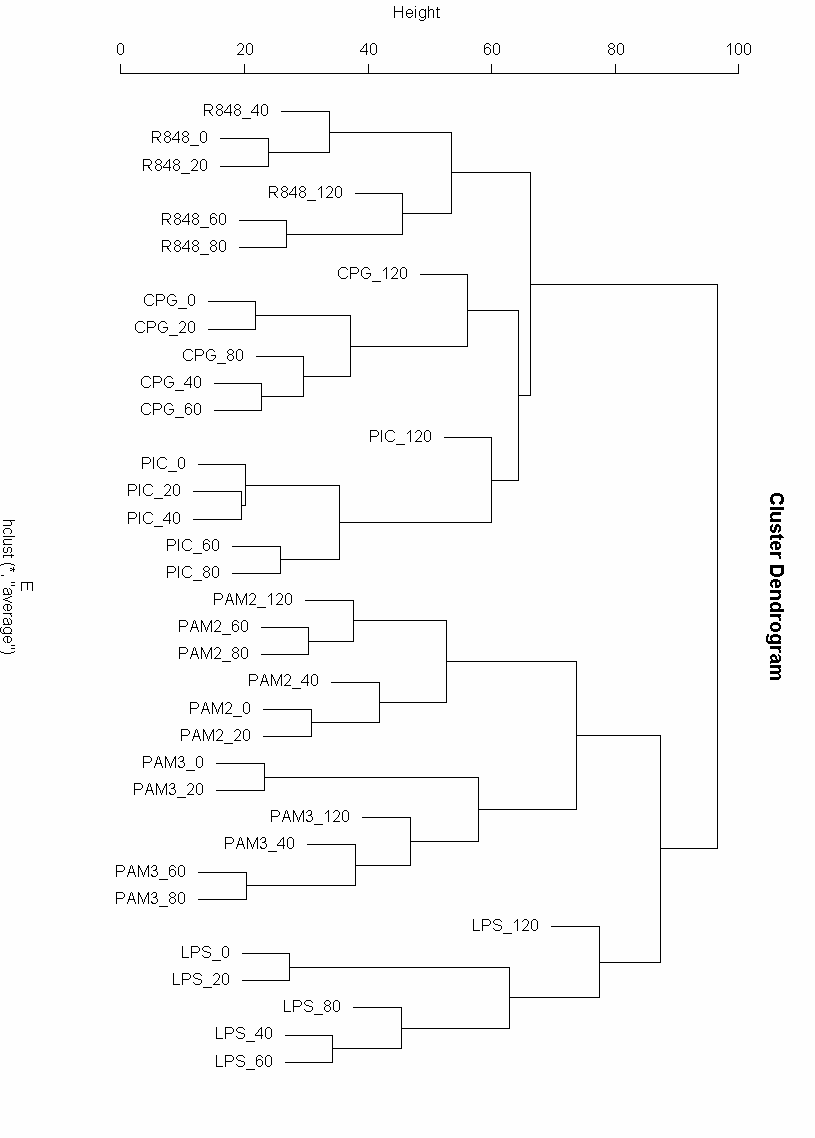

Supplement: Additional file 3 — Simulation data, results and scripts. Contains the script that generated the virtual transcript enumeration data, the dataset used as the basis for the analysis, the results from it, and the conclusions for all tested samples sizes n from 100,000 to 100,000,000. [file 1471-2105-8-246-S3.zip › Exp_Affy_BMM_1.txt.average.png]

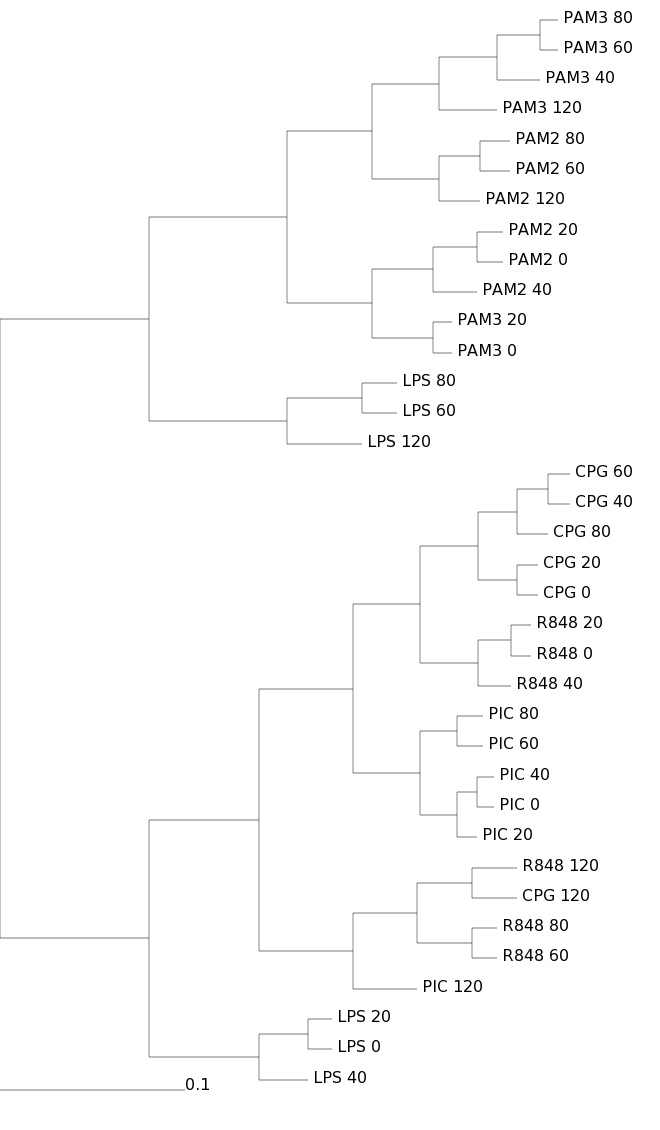

Supplement: Additional file 3 — Simulation data, results and scripts. Contains the script that generated the virtual transcript enumeration data, the dataset used as the basis for the analysis, the results from it, and the conclusions for all tested samples sizes n from 100,000 to 100,000,000. [file 1471-2105-8-246-S3.zip › Exp_Affy_BMM_N=1E5-average.png]

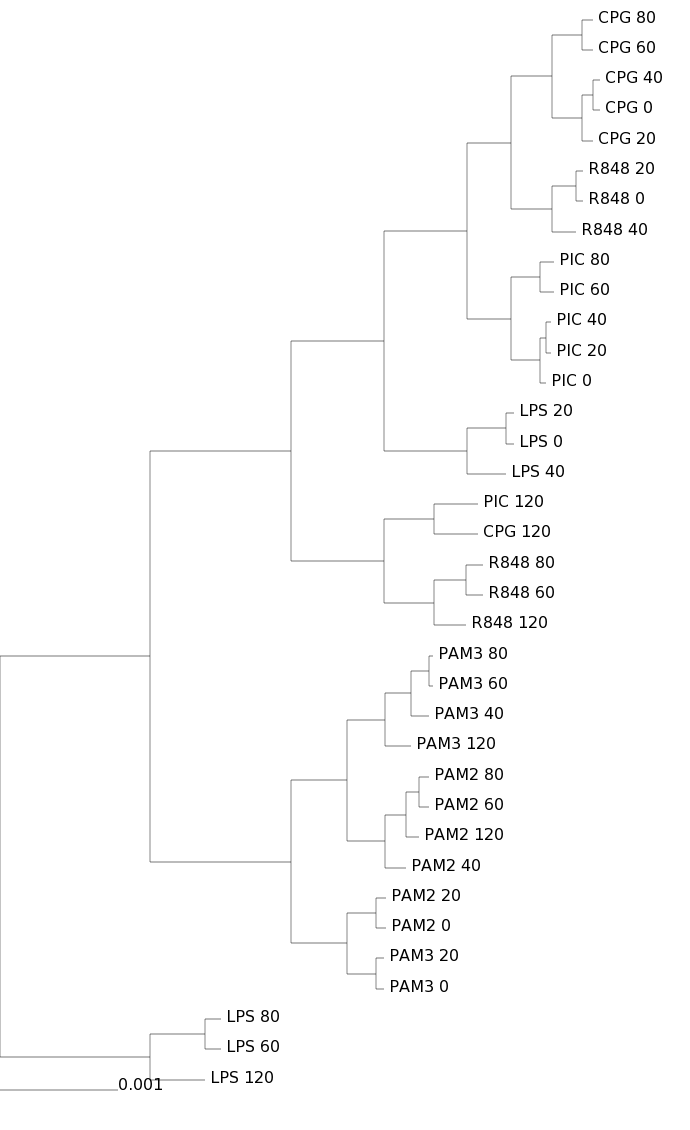

Supplement: Additional file 3 — Simulation data, results and scripts. Contains the script that generated the virtual transcript enumeration data, the dataset used as the basis for the analysis, the results from it, and the conclusions for all tested samples sizes n from 100,000 to 100,000,000. [file 1471-2105-8-246-S3.zip › Exp_Affy_BMM_N=1E5-correlation-average.png]

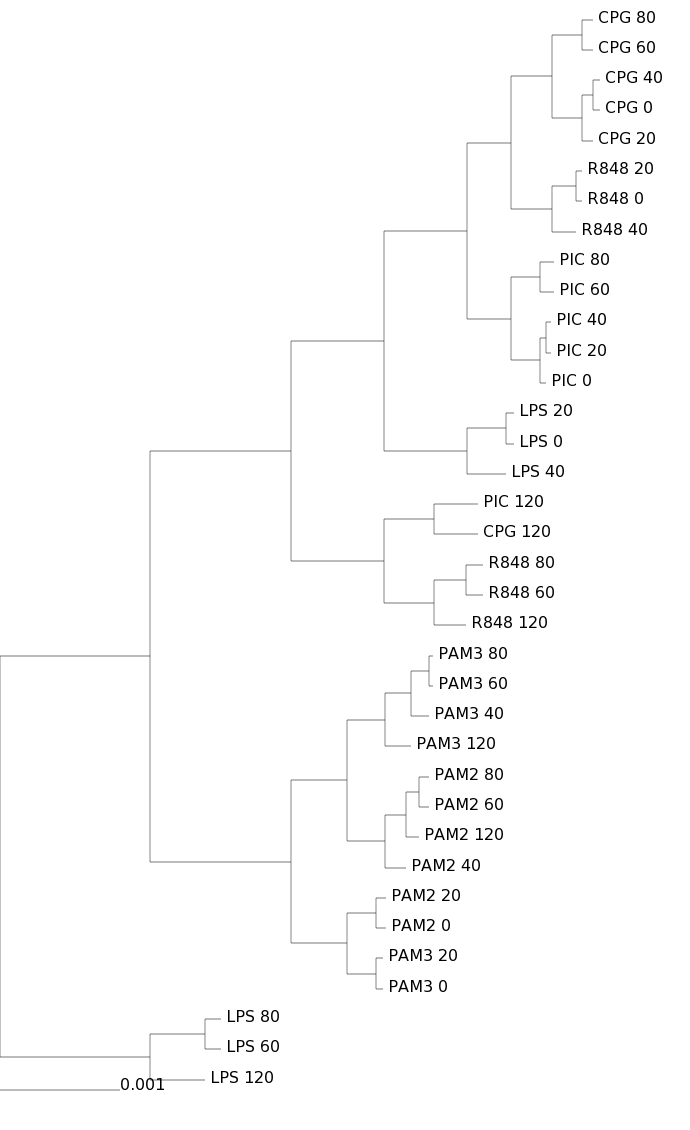

Supplement: Additional file 3 — Simulation data, results and scripts. Contains the script that generated the virtual transcript enumeration data, the dataset used as the basis for the analysis, the results from it, and the conclusions for all tested samples sizes n from 100,000 to 100,000,000. [file 1471-2105-8-246-S3.zip › Exp_Affy_BMM_N=1E5-cosine-average.png]

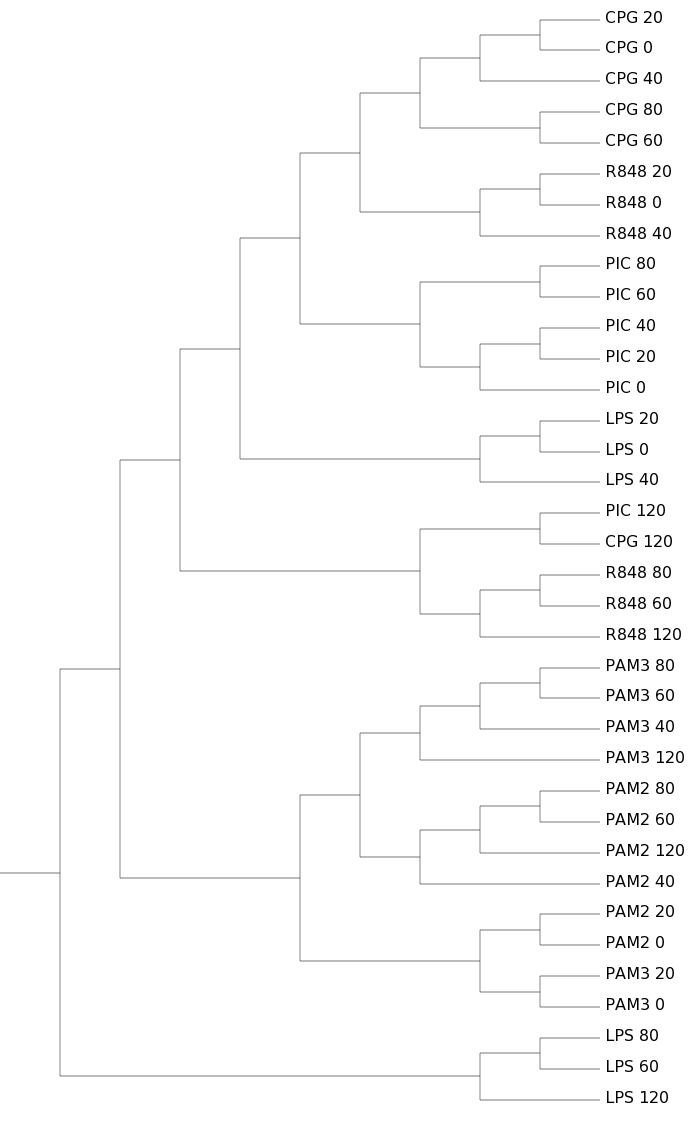

Supplement: Additional file 3 — Simulation data, results and scripts. Contains the script that generated the virtual transcript enumeration data, the dataset used as the basis for the analysis, the results from it, and the conclusions for all tested samples sizes n from 100,000 to 100,000,000. [file 1471-2105-8-246-S3.zip › Exp_Affy_BMM_N=1E5-euclid-average.png]

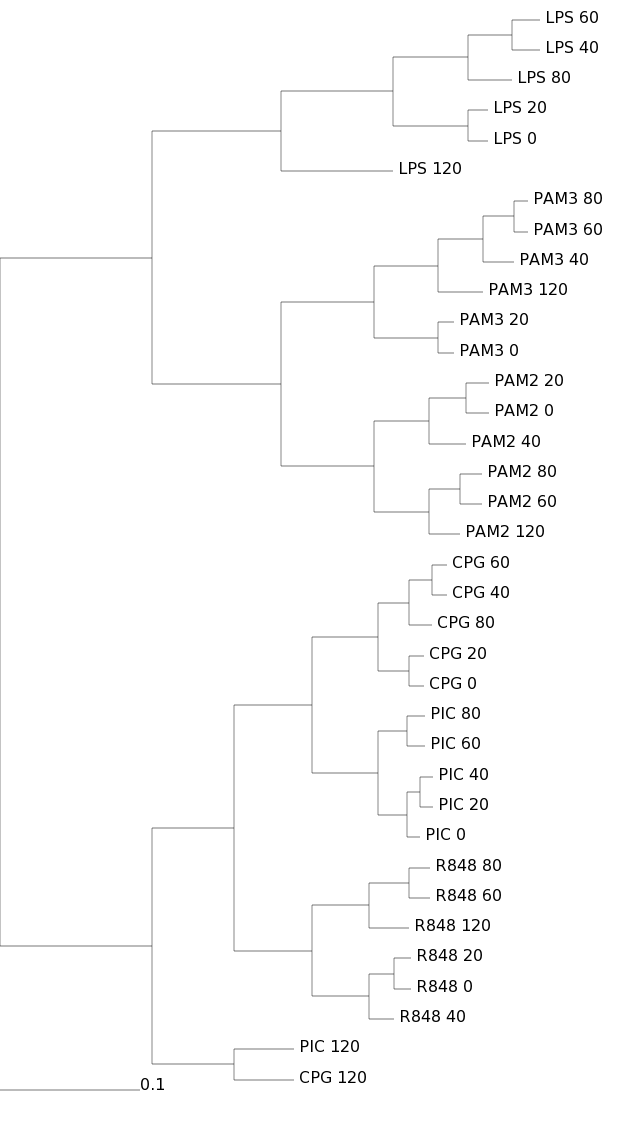

Supplement: Additional file 3 — Simulation data, results and scripts. Contains the script that generated the virtual transcript enumeration data, the dataset used as the basis for the analysis, the results from it, and the conclusions for all tested samples sizes n from 100,000 to 100,000,000. [file 1471-2105-8-246-S3.zip › Exp_Affy_BMM_N=1E6-average.png]

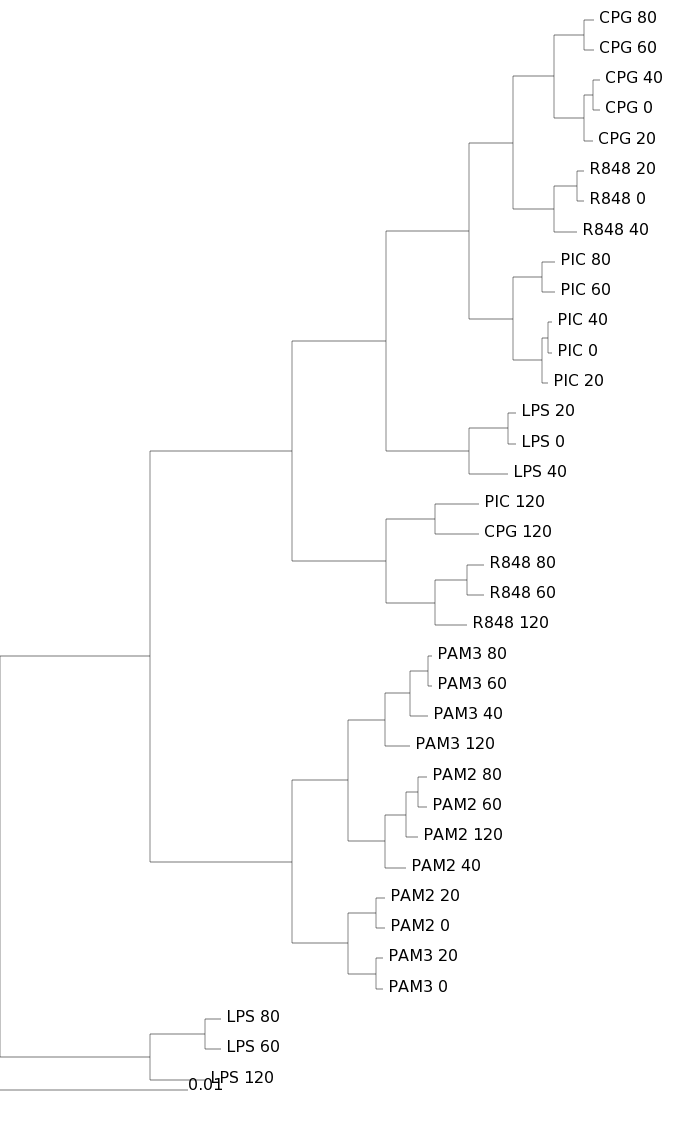

Supplement: Additional file 3 — Simulation data, results and scripts. Contains the script that generated the virtual transcript enumeration data, the dataset used as the basis for the analysis, the results from it, and the conclusions for all tested samples sizes n from 100,000 to 100,000,000. [file 1471-2105-8-246-S3.zip › Exp_Affy_BMM_N=1E6-correlation-average.png]

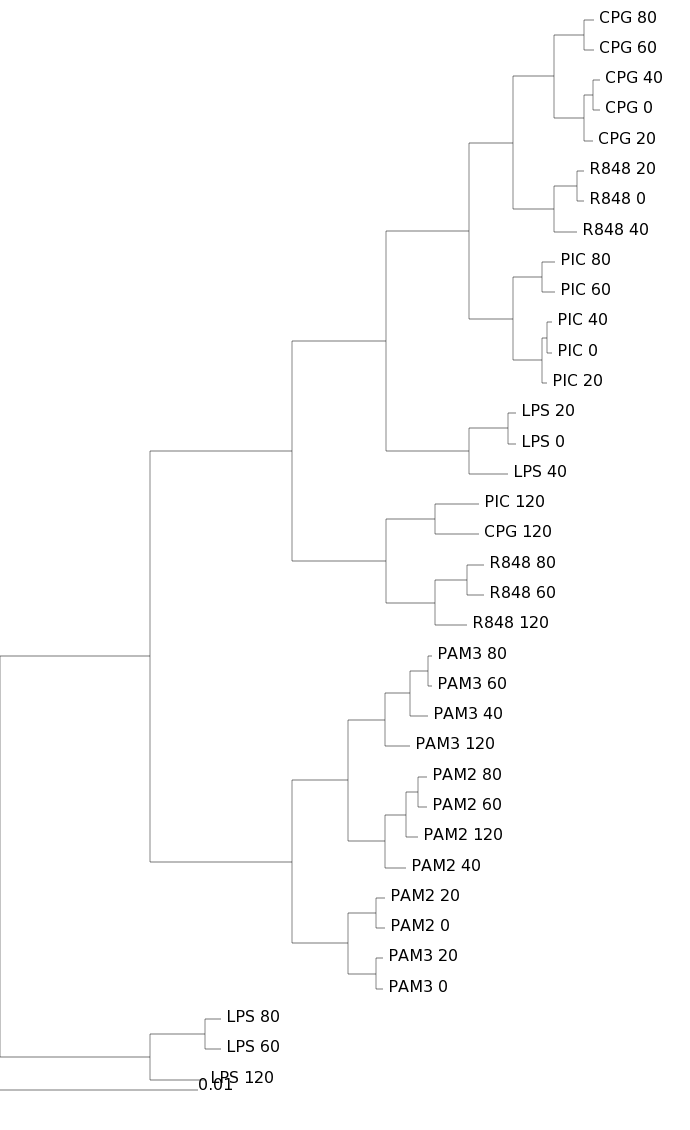

Supplement: Additional file 3 — Simulation data, results and scripts. Contains the script that generated the virtual transcript enumeration data, the dataset used as the basis for the analysis, the results from it, and the conclusions for all tested samples sizes n from 100,000 to 100,000,000. [file 1471-2105-8-246-S3.zip › Exp_Affy_BMM_N=1E6-cosine-average.png]

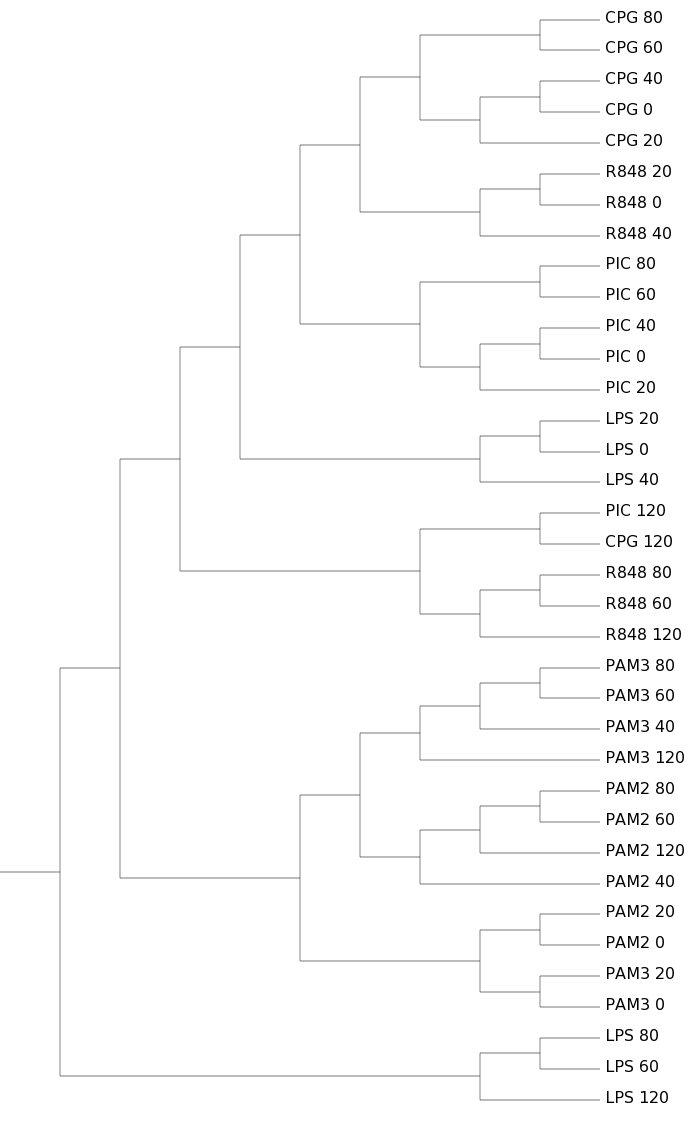

Supplement: Additional file 3 — Simulation data, results and scripts. Contains the script that generated the virtual transcript enumeration data, the dataset used as the basis for the analysis, the results from it, and the conclusions for all tested samples sizes n from 100,000 to 100,000,000. [file 1471-2105-8-246-S3.zip › Exp_Affy_BMM_N=1E6-euclid-average.png]

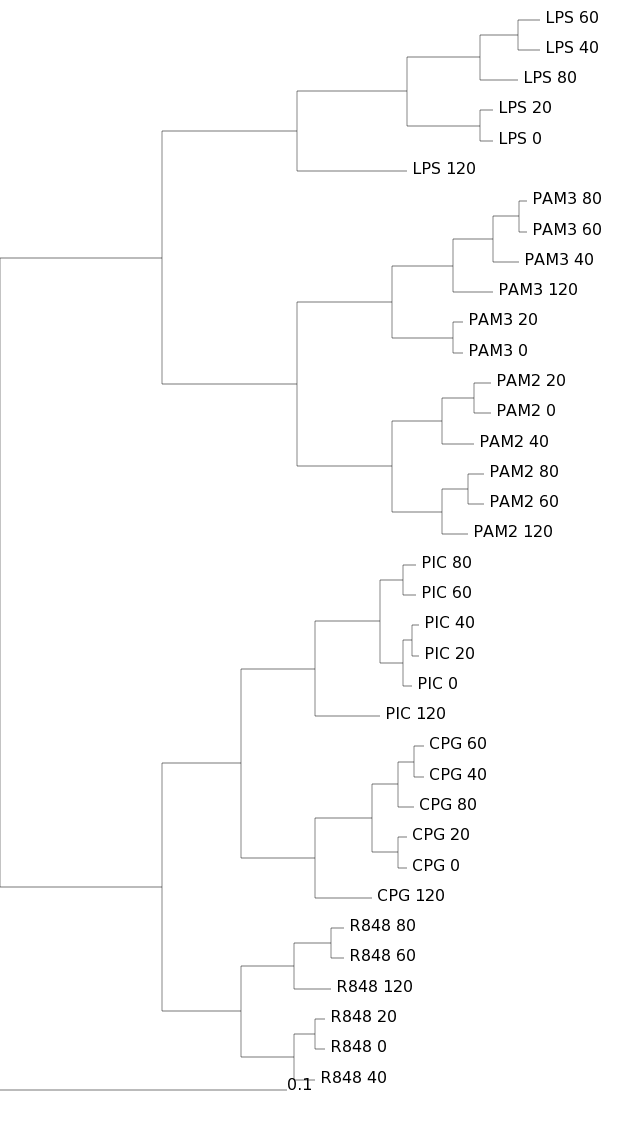

Supplement: Additional file 3 — Simulation data, results and scripts. Contains the script that generated the virtual transcript enumeration data, the dataset used as the basis for the analysis, the results from it, and the conclusions for all tested samples sizes n from 100,000 to 100,000,000. [file 1471-2105-8-246-S3.zip › Exp_Affy_BMM_N=1E7-average.png]

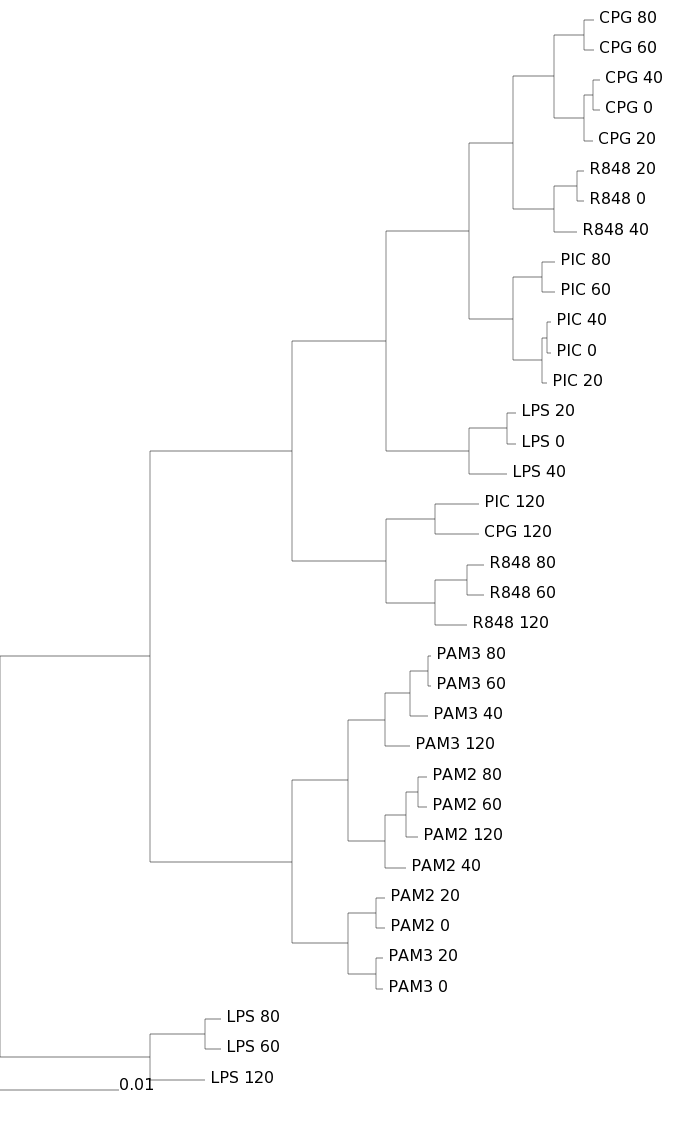

Supplement: Additional file 3 — Simulation data, results and scripts. Contains the script that generated the virtual transcript enumeration data, the dataset used as the basis for the analysis, the results from it, and the conclusions for all tested samples sizes n from 100,000 to 100,000,000. [file 1471-2105-8-246-S3.zip › Exp_Affy_BMM_N=1E7-correlation-average.png]

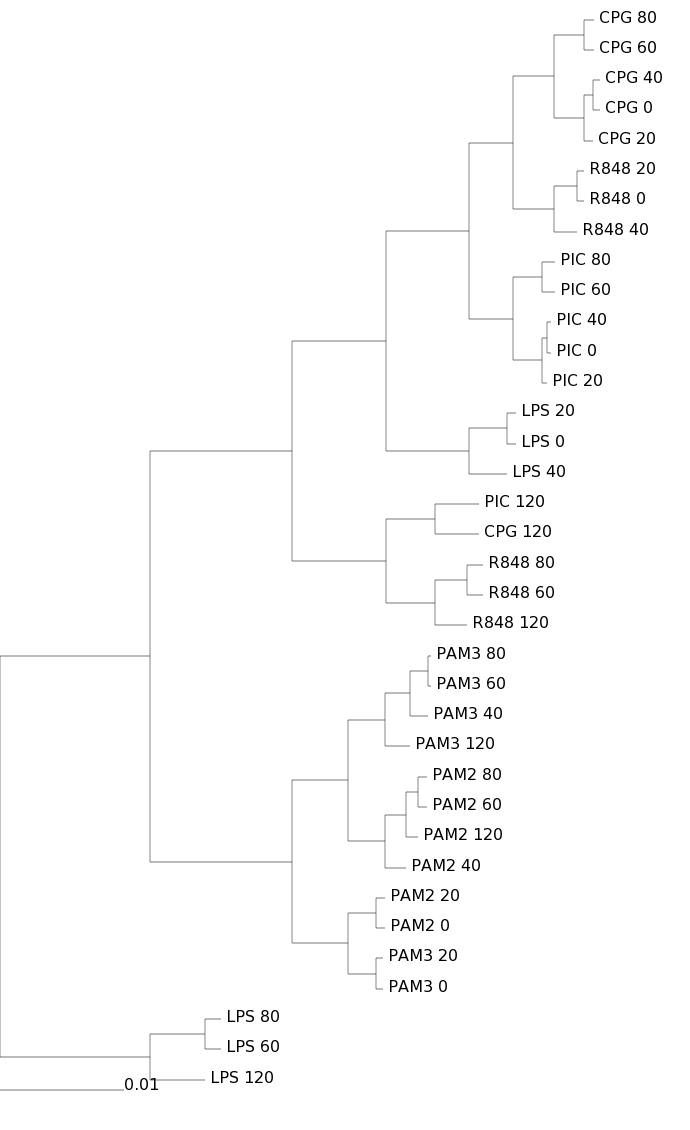

Supplement: Additional file 3 — Simulation data, results and scripts. Contains the script that generated the virtual transcript enumeration data, the dataset used as the basis for the analysis, the results from it, and the conclusions for all tested samples sizes n from 100,000 to 100,000,000. [file 1471-2105-8-246-S3.zip › Exp_Affy_BMM_N=1E7-cosine-average.png]

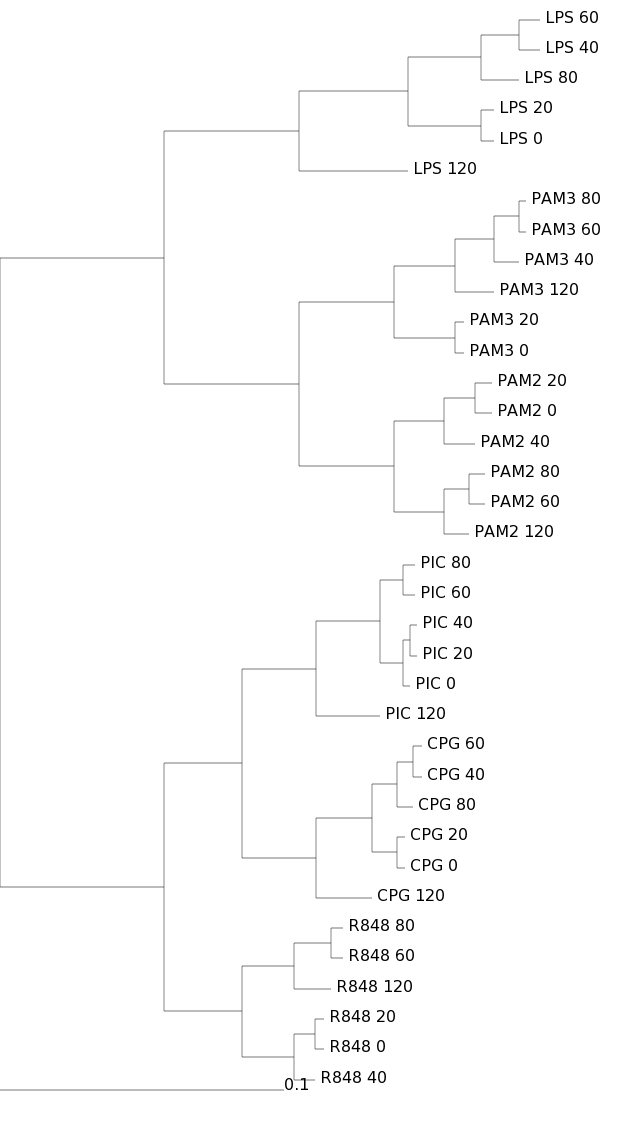

Supplement: Additional file 3 — Simulation data, results and scripts. Contains the script that generated the virtual transcript enumeration data, the dataset used as the basis for the analysis, the results from it, and the conclusions for all tested samples sizes n from 100,000 to 100,000,000. [file 1471-2105-8-246-S3.zip › Exp_Affy_BMM_N=1E8-average.png]

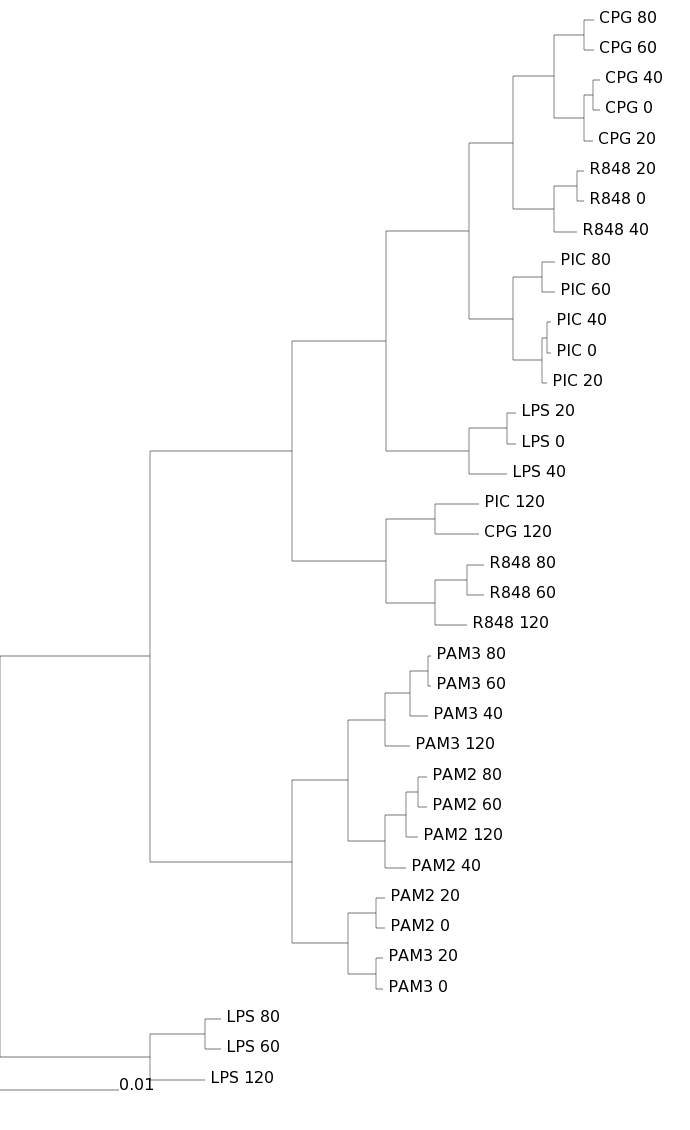

Supplement: Additional file 3 — Simulation data, results and scripts. Contains the script that generated the virtual transcript enumeration data, the dataset used as the basis for the analysis, the results from it, and the conclusions for all tested samples sizes n from 100,000 to 100,000,000. [file 1471-2105-8-246-S3.zip › Exp_Affy_BMM_N=1E8-correlation-average.png]

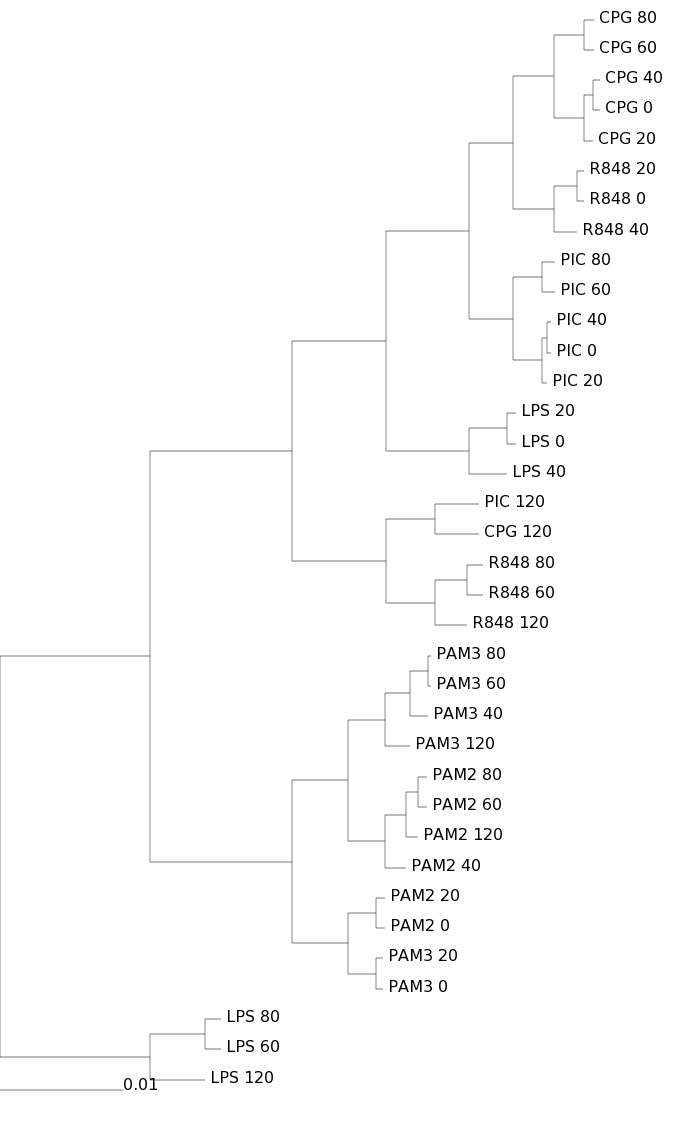

Supplement: Additional file 3 — Simulation data, results and scripts. Contains the script that generated the virtual transcript enumeration data, the dataset used as the basis for the analysis, the results from it, and the conclusions for all tested samples sizes n from 100,000 to 100,000,000. [file 1471-2105-8-246-S3.zip › Exp_Affy_BMM_N=1E8-cosine-average.png]

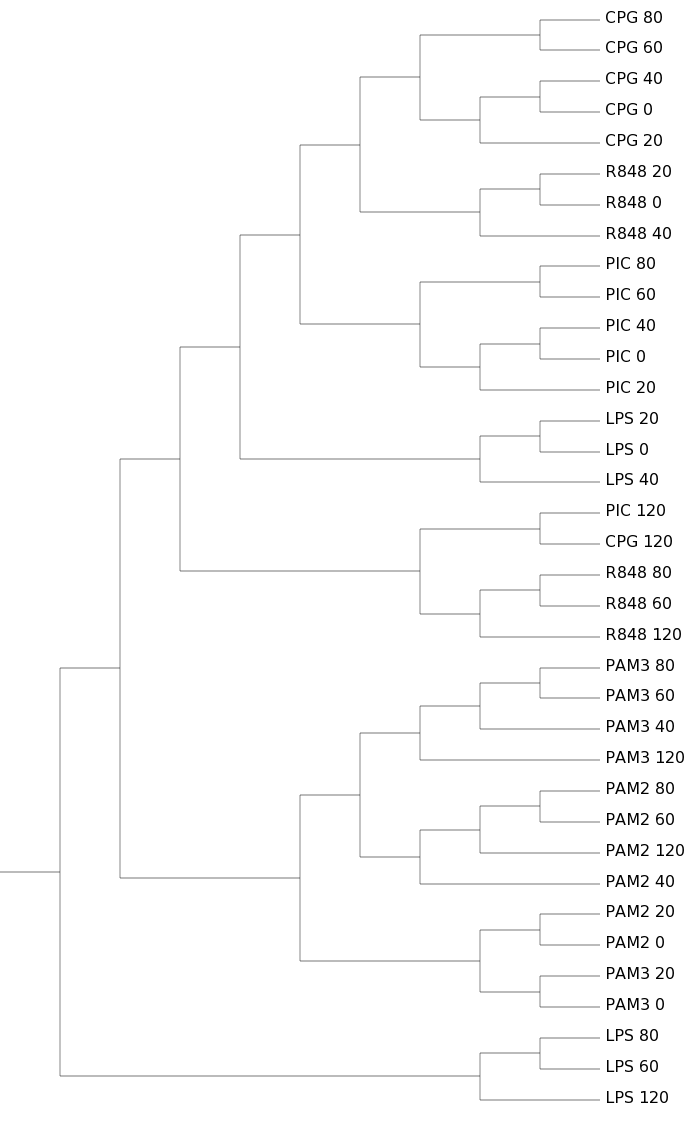

Supplement: Additional file 3 — Simulation data, results and scripts. Contains the script that generated the virtual transcript enumeration data, the dataset used as the basis for the analysis, the results from it, and the conclusions for all tested samples sizes n from 100,000 to 100,000,000. [file 1471-2105-8-246-S3.zip › Exp_Affy_BMM_N=1E8-euclid-average.png]
